# Comparison of freehand technique and a novel laser guiding navigation in distal locking of femoral intramedullary nails: a randomized controlled trial

**DOI:** 10.1186/s12893-022-01815-5

**Published:** 2022-10-21

**Authors:** Hua Gao, Zhenyu Liu, Xiaodong Bai, Guoqiang Xu, Wentao Chen, Ji Ma, Yijun Wang, Jiatian Wang, Gang Wang, Baojun Wang

**Affiliations:** Department of Orthopaedics, Beijing Friendship Hospital, Capital Medical University, Beijing, China

**Keywords:** Femoral fracture, Intramedullary nail, Distal locking, Laser guiding navigation device, Freehand technique

## Abstract

**Background:**

Intramedullary nail (IMN) is one of the key essential minimally invasive “weapons” in orthopaedic trauma, while the distal locking is still challenging for surgeons. Although there are various inventions and technologies to improve the locking procedure, there are still problems such as inaccurate positioning, excessive radiation exposure, low first success rate and long learning curve. Therefore, a new laser guiding navigation device was designed and compared with the traditional freehand (FH) technique in the distal locking of femoral IMN.

**Methods:**

This randomized controlled single-blind trial recruited patients with femoral diaphyseal fracture. The self-designed laser navigation device (laser group) and freehand technique (FH group) were used in the distal locking of the IMNs. The patients enrolled were randomized into FH group and laser group, all operations were performed by two surgeons of the same level. The differences between the two groups were compared in terms of radiation exposure time, operative time, first success rate, blood loss, visual analogue score (VAS), Harris score and healing time.

**Results:**

32 patients ended the study period and 16 patients in each group. The results showed that the laser group was better than the FH group in terms of distal locking time (10(9/11) vs 19.5 (17.25/21) min, *Z* = 4.83, *P* < 0.001), distal locking radiation exposure time (46.5 (41.25/51.75) vs 105 (88.25/140) s, *Z* = 4.807, *P* < 0.001), first success rate (30/32 vs 20/32, *χ*^2^ = 9.143, *P* = 0.002) and blood loss (60 (50–100) vs 150 (105–192.5) mL, *Z* = 3.610, *P* = 0.0003). There was no difference in Harris score, VAS score, or fracture healing time between the two groups.

**Conclusion:**

Compared with the FH technique, the novel laser guiding navigation device for distal locking of femoral IMN has the advantages of shorter operative time, less radiation exposure and higher first success rate.

*Trial registration* Chinese Clinical Trial Registry, ChiCTR2200060236. Registered 23 May 2022, https://www.chictr.org.cn/showprojen.aspx?proj=169130

## Introduction

Intramedullary nailing is one of the current standard treatments for long bone diaphyseal fractures [1–3], however, positioning the distal locking screw is one of the greatest challenges [4–7], especially for novice surgeons. Many novel technologies have been invented to optimize the locking procedure, such as electromagnetic navigation technology, computer-aided navigation technology, orthopedic robots and so on [8–18]. However, these technologies still cannot meet the needs of accuracy, simplicity and low radiation exposure [19].

Our team put forward the idea of visualizing the target screw passage. According to the basic principle of point, line and plane, we proposed the method of “three-line coaxial positioning” [20], combined the G-arm machine and laser, and designed a laser guiding navigation device, which had been patented. This device has been proved to be accurate, simple, and easy to master through the preliminary in vitro experiment. This study was approved by the ethics committee of Beijing Friendship Hospital (No. 2021-P2-029-02) to verify its safety and effectiveness in patients with femoral fracture treated with IMN fixation.

## Materials and methods

This was a randomized controlled single-blind trial. Subjects were recruited in Beijing Friendship Hospital. Inclusion criteria were male or female above 18 years of age, close fracture, acute fracture (The time between operation and injury was less than 2 weeks), subjected to IMNs (WASTON, Professional X Series) and patients had given written informed consent. Exclusion criteria were pathological fracture, old fracture, open fracture, secondary surgery and patients preferred locking plates. The patients enrolled were randomized into FH group and laser group. Randomization seed was specified and the randomization sequence was generated by an independent statistician utilizing the PROC PLAN procedure of SAS version 9.4 software with a 1:1 allocation. Two lists of random numbers (0–30) in sealed envelopes were concealed from the patients at all times. The operating surgeon selected from the sealed envelopes on the morning of the surgery to obtain the randomization allocation to treatment. All operations were performed by two senior attending physicians who received the same amount of time training. Each of them completed more than 30 femoral IMN operations by themselves with freehand technique in 2 years and their skill levels were very close.

The patients were placed in the supine position on a fracture traction table and underwent the same surgical procedure with antegrade nailing position except the distal locking process. In the FH group, the first step was to make the distal nail hole appear as a perfect circle in the lateral image of the G-arm image intensifier (G-arm Orca, WHALE MEDCINE, Boston, USA). Then the Kirschner wire (Φ = 2 mm) was gradually drilled along the same axis under fluoroscopic guidance. After confirming that the Kirschner wire passed through the target nail hole through the anterior–posterior and lateral images of the G-arm machine, the Kirschner wire was pulled out, the surgeon used a drill bit (4 mm) to enlarge the screw passage along the entry point and direction of the Kirschner wire, measured the length, and inserted the locking screw (5 mm). The second distal locking screw was inserted in the same way as above.

**Fig. 1:**
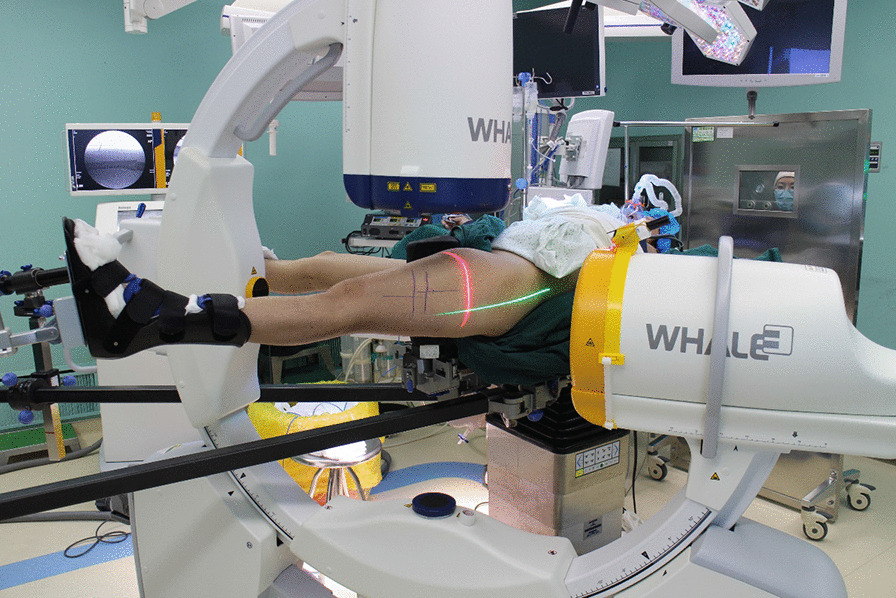
The physical photos of the laser guiding navigation device. The horizontal green-light laser pointer and the coronal red-light laser pointer were fixed to the G-arm’s image intensifier through the yellow round strap

The laser guiding navigation device is composed of a horizontal green-light laser pointer (in-line, energy 100 mW, wavelength 520 nm, Senwei), a coronal red-light laser pointer (in-line, energy 100 mW, wavelength 635 nm, Senwei), two laser pointer pedestals and a round strap attached to the G-arm’s image intensifier (Fig. 1). Make sure the intersection of the two laser lines, X-ray fluoroscopy center and display screen center were completely overlapped through preoperative adjustment.

**Fig. 2:**
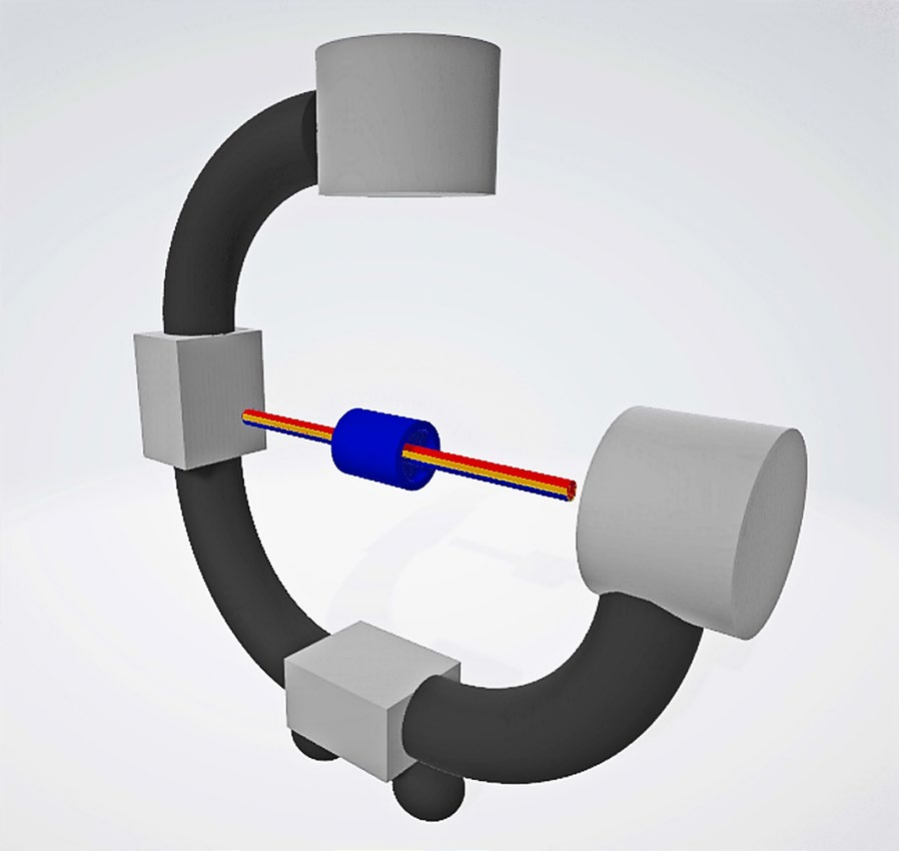
Work principle sketch maps of the device. This meant that the X-ray central fluoroscopy axis (the yellow line), the two laser lights cross axis (the red line) and the target screw passage (the blue line) remain coaxial, known as “three-line coaxial”. The blue tube represented a nail hole

**Fig. 3:**
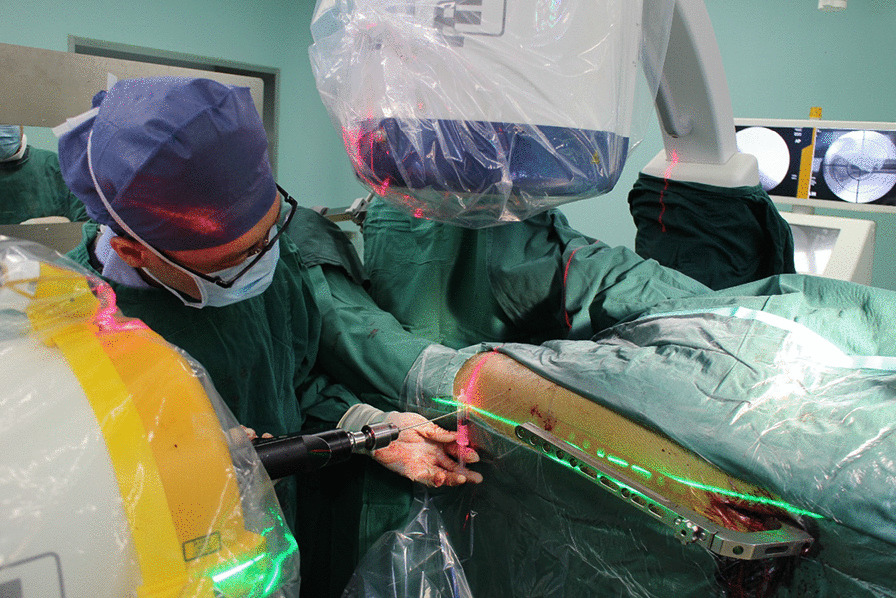
The surgeon dilled the Kirschner wire (Φ = 2 mm) along the red and green laser lines. A plastic syringe was used to adjust and control the direction of the Kirschner wire

**Fig. 4:**
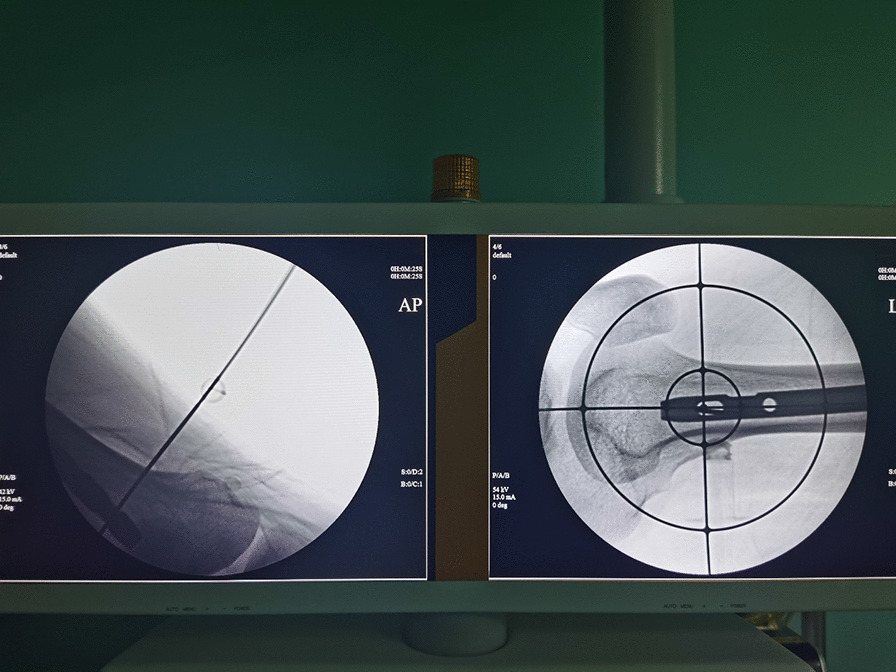
X-ray fluoroscopy confirmed that the Kirschner wire passed through the nail hole

In the laser group, the first step was to make the distal nail hole appear as a perfect circle in the lateral image as in the FH group. Then the G-arm was moved to ensure that the center of the nail hole was overlapped with the display screen center. This meant that the X-ray central fluoroscopy axis (the display screen center), the two laser lights cross axis and the target screw passage remain coaxial, known as “three-line coaxial” (Fig. 2). The surgeon drilled the Kirschner wire (Φ = 2 mm) along the red and green laser lines (Fig. 3). X-ray fluoroscopy confirmed that the Kirschner wire passed through the nail hole (Fig. 4). The remaining surgical procedures were the same as FH group and the second distal locking screw was inserted in the same way.

The first success rate, distal locking time, distal locking radiation exposure time, amount of bleeding, postoperative visual analogue score (VAS), Harris score and healing times were recorded and used for comparison in two groups. The patients were followed up at 1, 2, 3 and 6 months postoperatively, or the follow-up ended after fracture healing.

According to the data obtained in the preliminary vitro experiment [20], the distal locking time (s) and distal locking radiation exposure time (s) were used as the main observation indicators for estimation. The distal locking time of the laser group and the FH group were 135 ± 5 s and 190 ± 39 s, respectively, and the distal locking radiation exposure time was 15 ± 3 s and 98 ± 14 s, respectively. We set the first-class error to 0.025 and the power to be 0.9 through calculation of PASS software based on the two main research indicators. The minimum sample size for the study of the distal locking time (s) was 16 and 6 for the distal locking radiation exposure time (s). Considering the actual situation of this study, the sample size of each group was finally determined to be 16.

The first success rate was presented as percentage and the paired Chi-square test was used to evaluate differences between two groups. Continuous data including the other parameters were tested for normal distribution. The variables of normal distribution were presented as mean ± standard deviation (SD), and the independent sample *t* test was used for statistical test. Variables that did not meet the normal distribution were described as median (upper/lower quartile) and the Wilcoxon rank-sum test was used for comparison between groups. All statistical analyses were performed using the SAS JMP version 16.0 software and statistical significance was defined as *P* < 0.05.

## Result

**Fig. 5:**
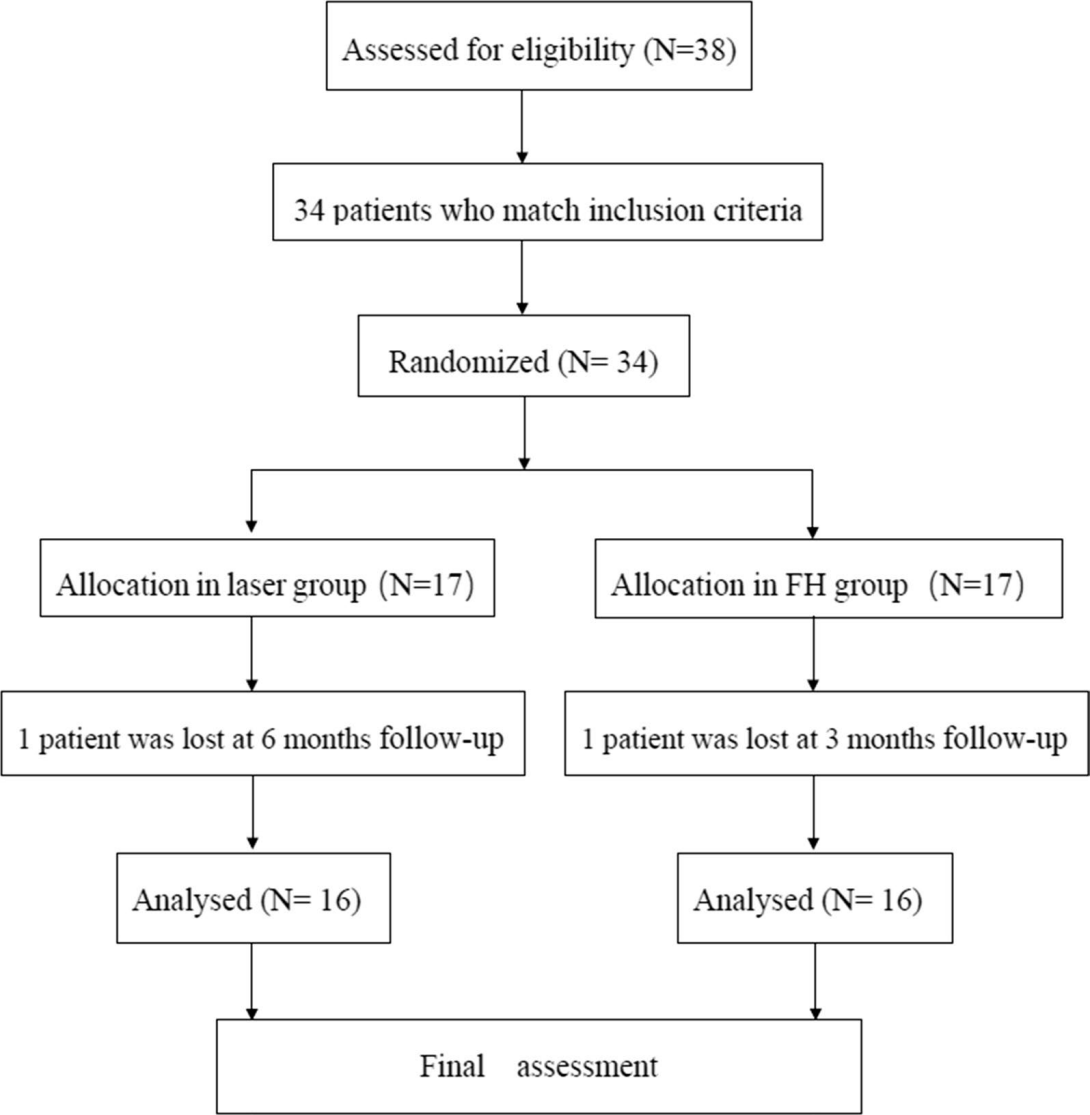
Flowchart of the study design

Of the 38 patients admitted to our hospital from June 2021 to November 2021, 34 patients who matched the inclusion and exclusion criteria were recruited (Fig. 5). 32 ended the study period. 16 patients were randomized to the FH group and 16 patients to the laser group. Reasons to drop out were lost at postoperative follow-up (two patients). The characteristics of patients in two groups was shown in Table 1. There was no statistically significant difference in age, gender, fracture localization and fracture classification.

**Table 1:** The characteristics of patients in two groups

	Age	Gender		Fracture localization		AO classification		
		Male	Female	Left	Right	32-A	32-B	32-C
Laser group (n = 16)	45.0 ± 6.2	10	11	7	9	11	2	3
FH group (n = 16)	44.9 ± 5.9	6	5	9	7	13	2	2
*t*/*χ*^2^ value	0.047	0.139		0.500		0.337		
*P* value	0.963	0.710		0.724		0.845		

**Fig. 6:**
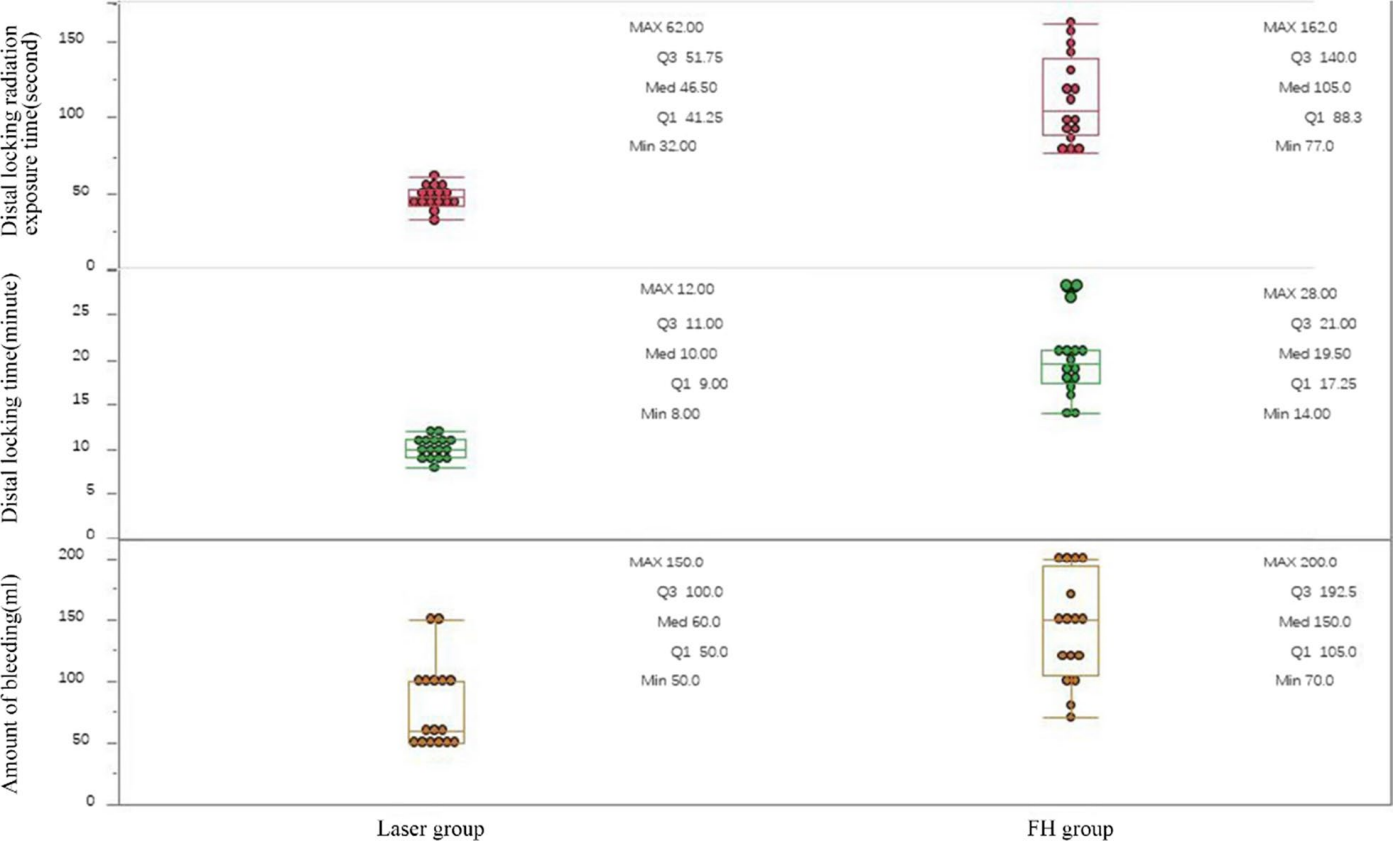
The box plot showed that the laser group was better than the FH group in terms of distal locking time, distal locking radiation exposure time and blood loss

The first success rate was 93.75% (30/32) in the laser group and 62.5% (20/32) in the FH group, the difference was statistically significant (χ^2^ = 9.143, *P* = 0.002). The distal locking time of the laser group was 10 (9/11) min, whereas the time was 19.5 (17.25/21) min in the FH group. The distal locking radiation exposure time of the laser group and the FH group was 46.5 (41.25/51.75) s and 105 (88.25/140) s respectively. The distal locking time of the laser group was statistically significantly shorter compared to the freehand group (*Z* = 4.83, *P* < 0.001), and the exposure time was significantly reduced in the laser group compared to the FH group (*Z* = 4.807*, P* < 0.001). The amount of bleeding of the laser group and the FH group were 60 (50/100) mL and 150 (105/192.5) mL respectively, and the difference was statistically significant (*Z* = 3.61, *P* = 0.0003) (Fig. 6). There was no statistical difference in VAS score, Harris score or fracture healing time between the two groups (Table 2).

**Table 2:** The results of two groups

Parameter	Laser group	FH group	*t*/*χ*^2^/Z value	*P* value
First success rate***	93.75% (30/32)	62.5% (20/32)	9.143	0.002
Distal locking time(min)*	10 (9/11)	19.5 (17.25/21)	4.83	< 0.0001
Distal locking radiation exposure time (s)*	46.5 (41.25/51.75)	105 (88.25/140)	4.807	< 0.0001
Amount of bleeding (mL)*	60 (50/100)	150 (105/192.5)	3.61	0.0003
VAS (before operation)*	8 (8/9)	8 (8/9)	0.202	0.8402
VAS (1 week after operation)*	2 (2/2)	2 (2/2.75)	1.222	0.2217
Harris score**	88.63 ± 2.16	87.25 ± 2.86	− 1.534	0.1354
Healing time (week)**	17.25 ± 1.65	17.31 ± 1.45	0.114	0.9102

^*^Non-normal continuous data was presented as median (lower/upper quartile), and the statistical result was Z value

^**^The normal continuous data was presented as mean ± standard deviation, and the statistical result was *t* value

^***^Categorical variables were presented as frequency and percentage, and the statistical result was *χ*^2^ value

## Discussion

Many novel technologies have been invented to optimize the locking procedure, however, they have the following drawbacks: complex and expensive equipment, inaccuracy and cumbersome operation (Table 3) [8, 11, 15, 20–23]. The freehand technique is still the ultimate option where these methods don’t work and can be regarded as the gold standard. Therefore, this study chose the freehand technique as the control.

**Table 3:** Summary of the published novel technologies in the locking procedure (2017–2021)

Article	Localization	Technology	Precision accuracy	First success rate	Distal locking time	Radiation exposure time
Panzica et al. [21]	Cadaver femur specimen	Robotic technique (BrainLAB VectorVision)	–	100% (20/20)	123.8 ± 37.5 s	6.5 ± 3.6 s
Han et al. [8]	Femoral fracture	Electromagnetic navigation system (SURESHOT)	–	–	6.1 ± 1.4 min	2.2 ± 1.1 s
Tao et al. [23]	Femoral shaft fracture	Core drill	–	95.24% (20/21)	497.19 ± 82.78 s	12.81 ± 2.64 s
Hsu et al. [22]	Femoral model	Passive or active (robot arm) assistive device	Errors of 2.2 mm in position and 3.19° in direction	100% (36/36)	60.2 ± 15.6 s	2 min
Gao et al. [11]	Cadaver femur specimen	Electromagnetic navigation system (TianXuan-MDTS)	Errors of 1.60 ± 0.20 mm in position and 3.10 ± 0.84° in direction	100% (6/6)	143.17 ± 18.27 s	–
Maleki et al. [15]	Tibia shaft fracture	Laser indicator	–	100% (3/3)	115.333 ± 8.993 s	–
Gao et al. [20]	Femoral model	Laser guiding navigation device	Errors of 2.8 ± 0.5° in the coronal plane and 2.8 ± 0.5° in the horizontal plane	95% (38/40)	212 ± 105 s	41 ± 15 s

It takes 7–21 min to position the distal locking screw by freehand technique, which is technically challenging and under radiation exposure [22–25]. For novice surgeons, the situation can be even worse. Repeated manipulation can lead to cortical defect of lateral femur, reduce the firmness of the interlocking nail and cortex, lead to nail withdrawal, and even affect fracture healing [4, 26]. Improving the first success rate, shortening the operative time and reducing the radiation are technical problems of the distal locking of IMN.

The laser guiding navigation device is simple to install and only needs to be adjusted for the first time use before the operation, and it can be calibrated at any time during operation. A special positioning ring is used to locate the target screw passage, and then the guide pin is punched under the guidance of laser to achieve the effect of visualizing the target passage. Its accuracy has been verified by in vitro experiments [20].

**Fig. 7:**
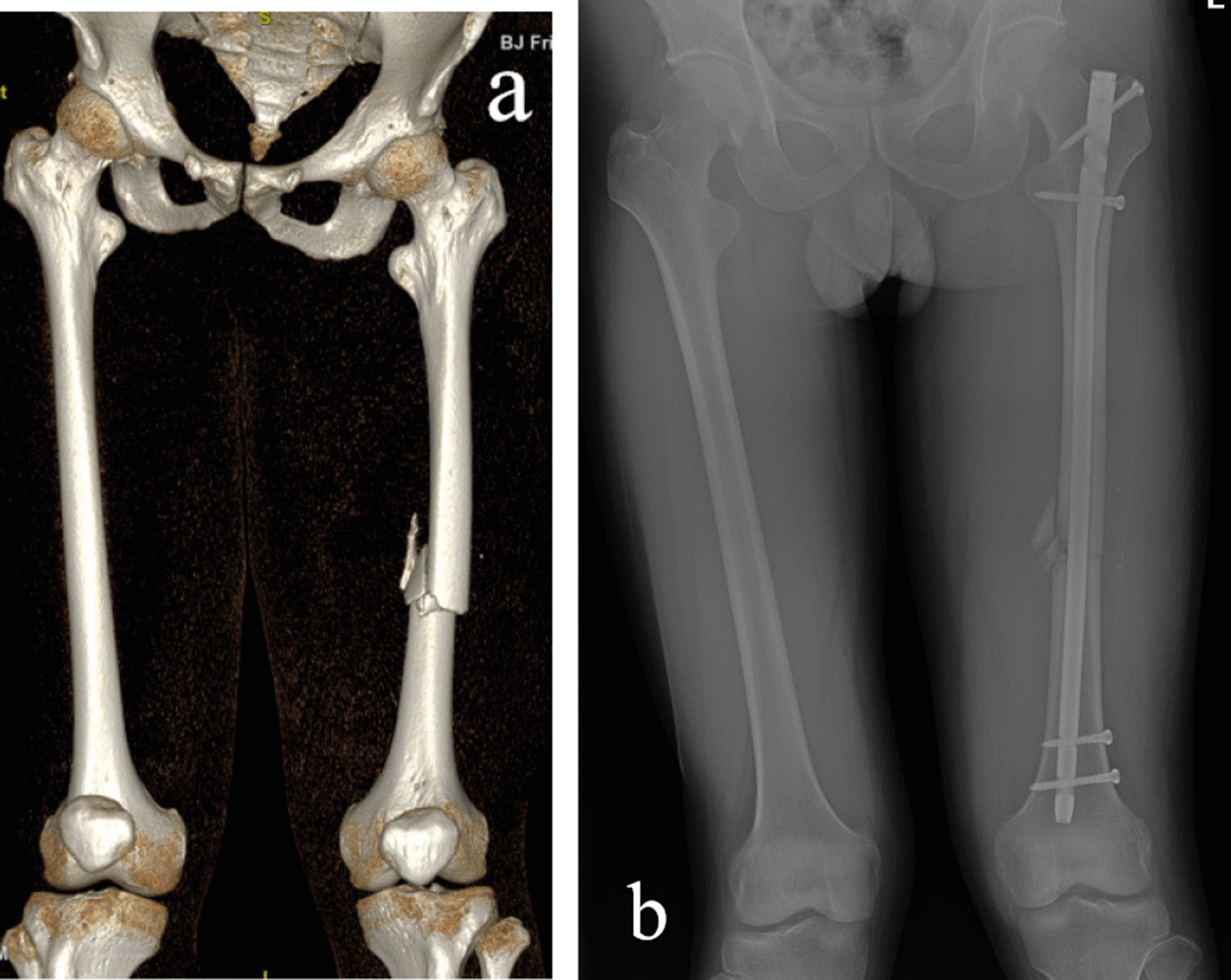
Preoperative three-dimensional CT image of a patient with femoral shaft fracture (**a**). X-ray image of this patient undertaken IMN with the laser guiding navigation device on the first day of postoperative (**b**)

In this study, there was no statistical difference in fracture healing time, postoperative VAS score and Harris score between the laser group and the FH group, indicating that femoral shaft fracture could be effectively treated and satisfactory efficacy achieved. In this study, the laser group performed better in terms of operative time, exposure time and intraoperative blood loss, which can improve the surgical efficiency. In this study, through clinical in vivo experiments, the first success rate of laser group can reach 93.75%, compared with the first success rate of freehand group (62.5%), which has a significant improvement, further verified the characteristics of its high accuracy, and provided a new idea for solving this technical problem (Fig. 7).

Although this study was persuasive, there were still some limitations. First of all, although the number of cases had reached the sample size required by statistics, multi-center studies with more cases would make the results more convincing. Secondly, the device could achieve accurate positioning, but how to maintain the stability of guide pin in the process of drilling needed to be further improved. Thirdly, although the level of the two surgeons was close, it was still impossible to rule out the influence caused by the difference in technical level. In addition, the evaluation for the damage of the laser to the surgeon’s eyes was ignored.

## Conclusion

The novel laser guiding navigation device is accurate and effective, can reduce the operation time and radiation exposure, and has a high first success rate, which has the value of clinical promotion.

## Data Availability

The datasets used and/or analyzed during the current study are available from the corresponding author on reasonable request.

## Abbreviations

IMN: Intramedullary nail
VAS: Visual analogue score
FH: Freehand
